# Ketone ester-what’s in a name? Ambiguity begets uncertainty

**DOI:** 10.3389/fphys.2023.1197768

**Published:** 2023-05-09

**Authors:** M. Todd King

**Affiliations:** Laboratory of Integrative Neuroscience, NIAAA, NIH, Rockville, MD, United States

**Keywords:** ketone esters, ketone ester nomenclature, ketone bodies, beta-hydroxybutyrate, acetoacetate

## Introduction

Consumption of a “ketone ester” drink has resulted in either a significant improvement (Cox et al., 2016) or a significant impairment (Leckey et al., 2017) in cycling time trial performance. Results from the Cox and Leckey testing prompted a commentary by (Stubbs et al., 2018) potentially explaining the observed differences in outcome. While both groups tested “ketone esters”, the Cox group tested (*R*) 3-hydroxybutyl (*R*) 3-hydroxybutyrate while the Leckey group tested (*R*,*S*) 1,3-butanediol acetoacetate diester. The structural differences are apparent in their more formal names. The bulk of literature reports to date, involve the study of these two compounds although three additional “ketone ester” molecules have, more recently, been described.

The study of ketone esters is not limited to the field of endurance athletics and physiology. Heart failure (Yurista et al., 2021), cognitive impairment (Newport et al., 2015), protection from ionizing radiation (Curtis et al., 2016), cancer treatment (Seyfried et al., 2011), sepsis (Weckx et al., 2022) and lifespan (Veech et al., 2017) are among research areas where the study of ketone esters has garnered interest. In addition to their function as an alternative fuel to glucose, in most organs, for the purpose of generating ATP, ketone bodies, particularly βHB have many signaling and epigenetic contributions (Newman and Verdin, 2017). Similarities, and differences, in exogenous ketones, and their metabolites, have been neatly summarized by (Evans et al., 2022), (Poff et al., 2020), and (Falkenhain et al., 2022) among others.

Given the breadth of research interest, synthesis of novel “ketone esters” is certain to continue as research outcomes more clearly target specific chemical structures. Esters offer a convenient means of elevating circulating ketone levels without a concomitant acid or salt load. However, inclusion of generic terms, such as “ketone ester”, while brief and structurally correct, sow confusion in the minds of readers, particularly for those considering their use. The number of offerings begs for a simplified means of identifying each compound, to accompany the standard nomenclature, *in lieu* of “ketone ester”, much as NAD is understood to be shorthand for nicotinamide adenine dinucleotide.

One method of identification may be to reference a patent number wherein the compound is described. Using patent numbers, while brief, are problematic as patents are written broadly, and in a deliberately obfuscatory manner, in order to describe a host of related compounds, thus defeating the purpose of clear identification of individual molecules.

A second abbreviated naming option would be to reference, in some manner, the first report of a molecule’s use in the published literature. This option, while possibly not properly crediting the “inventor” would at least acknowledge the first published use of the molecule.

Yet another option would be to form a standing committee, or commission, of researchers invested in the field, established for the purpose of formulating a set of naming rules or guidelines that would result in the clear and unambiguous assignment of “ketone ester” names. This process would yield a short, unique identifier, much like a barcode or QR code for each compound that could be used in manuscripts in place of the vague term “ketone ester”. Such a code could be scannable on a personal electronic device and show the structure, with stereochemistry where appropriate, and include the IUPAC name.

Alternatively authors could submit the name(s) and structure(s) of a newly formulated compound(s) to said committee or commission which would then assign a short descriptor much as Chemical Abstracts Service assigns CAS numbers or the Enzyme Commission assigns EC numbers. It would be appropriate for such a committee to have standing in, or be recognized by, a professional society such as ACS or IUBMB. For example, such an IUPAC/IUBMB committee exists for establishing rules for the nomenclature of carbohydrates (McNaught, 1996).

Subsequent use of this short identifier should lessen confusion within the field regarding which specific compound was studied versus use of the generic term “ketone ester”.

**FIGURE 1:**
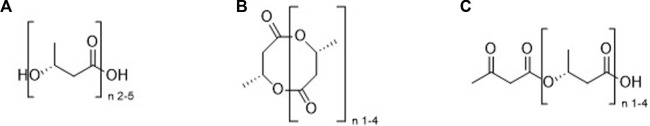
Oligomers of (*R*)-βHB; linear **(A)**, cyclic **(B)** or linear (*R*)-βHB containing a terminal AcAc moiety **(C)**.

**FIGURE 2:**
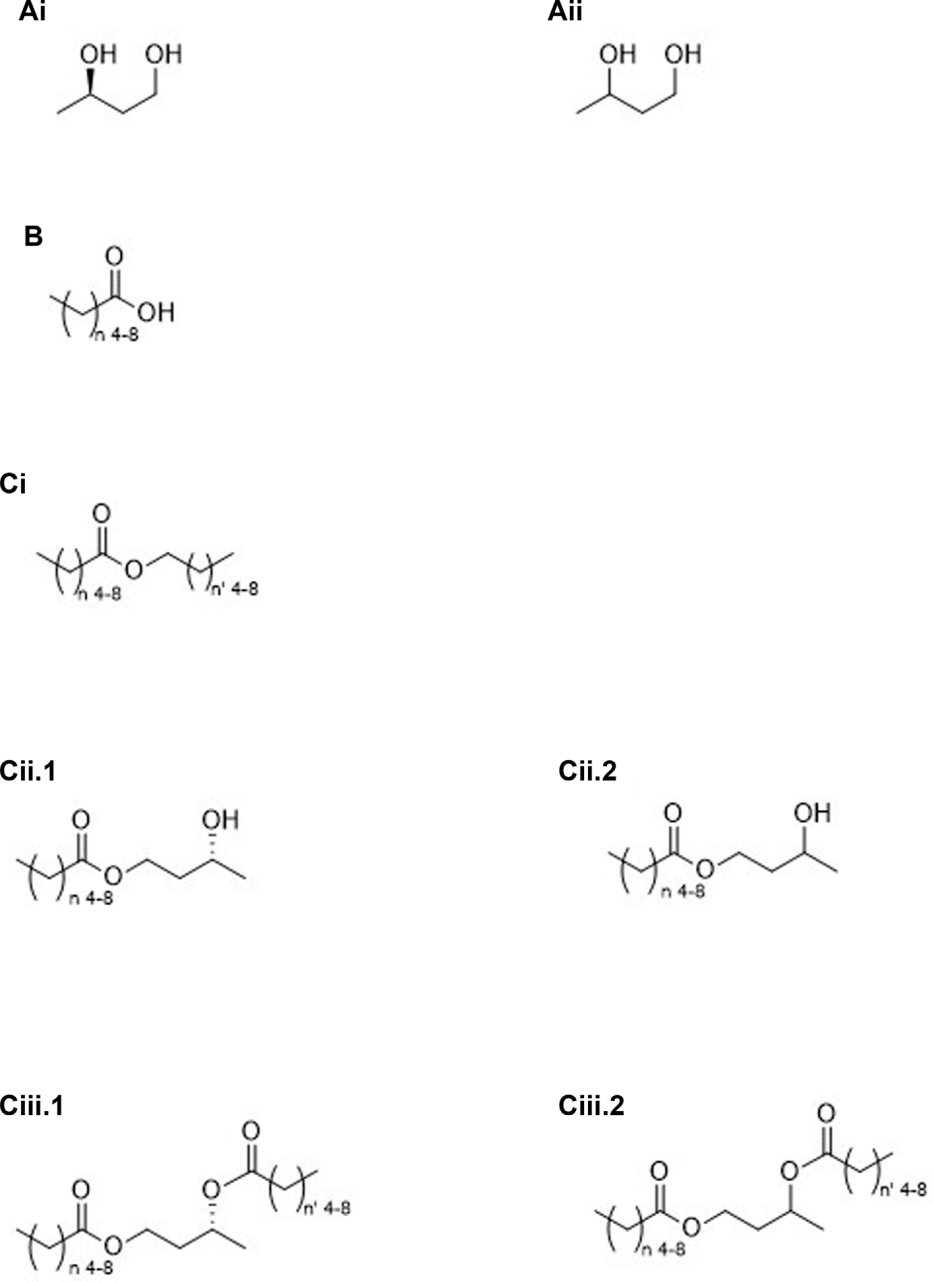
Molecules containing pro-ketone bodies: Group “A” (compounds converted to ketone bodies via NAD-linked oxidation) (*R*)-1,3-butanediol **(Ai)**, racemic 1,3-butanediol **(Aii)**; Group “B” (compounds converted to ketone bodies via β-oxidation), medium chain carboxylic acids **(B)**; Group “C” (esters converted to ketone bodies via both NAD-linked and β-oxidation), mid-chain alcohol condensed to a mid-chain carboxylic acid **(Ci)**, esters of mid-chain carboxylic acids and (*R*)-1,3-butanediol **(Cii.1)** or racemic 1,3-butanediol **(Cii.2)**, and diesters of mid-chain carboxylic acids and (*R*)-1,3-butanediol **(Ciii.1)** or racemic 1,3-butanediol **(Ciii.2)**.

**FIGURE 3:**
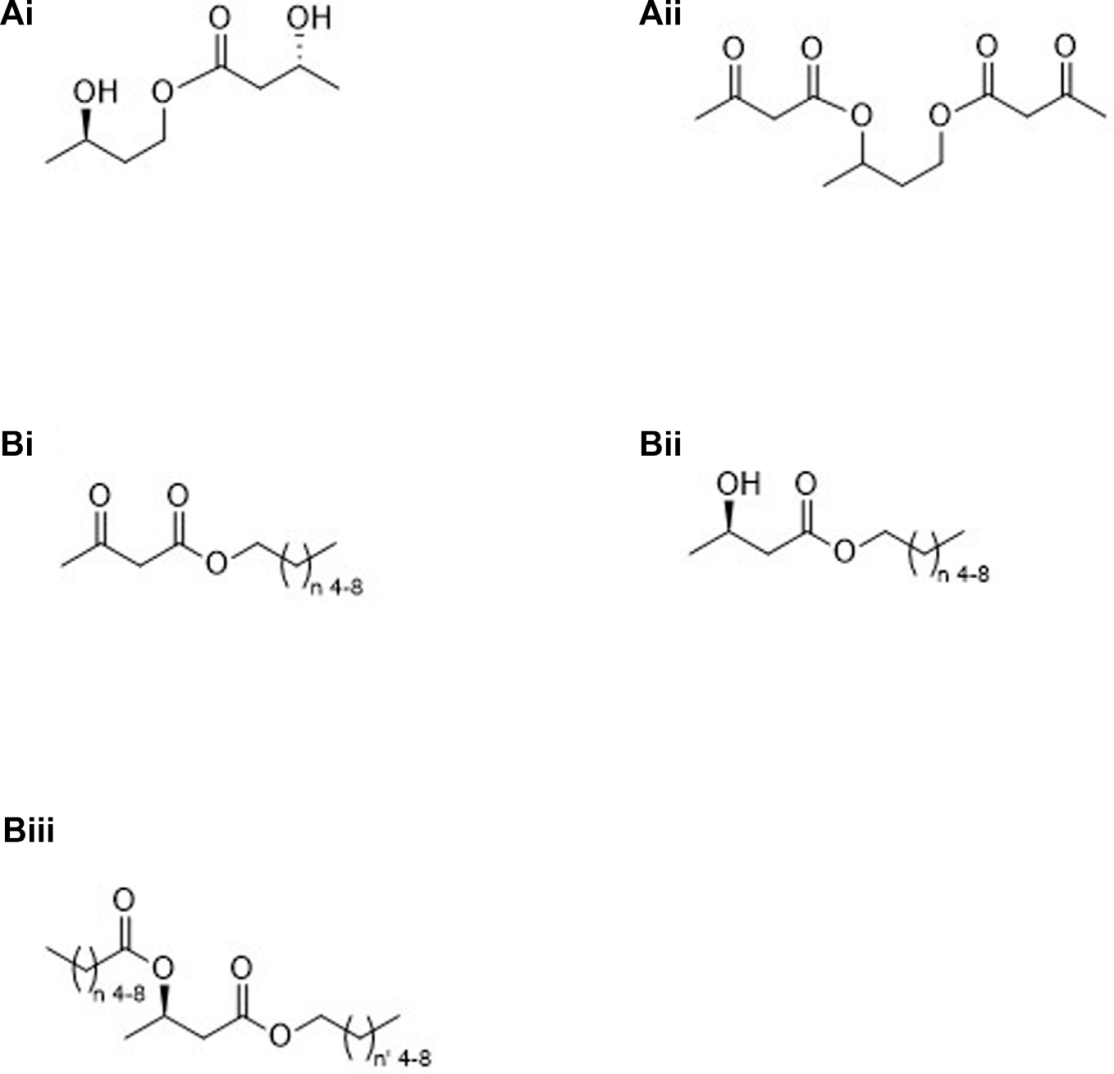
Molecules containing both pro-ketone bodies and ketone bodies: Group “A” (esters formed from a ketone body and either stereospecific or racemic 1,3-butanediol), (*R*)-3-hydroxybutyl (*R*)-3-hydroxybutyrate **(Ai)**, or 1,3-butanediol acetoacetate diester **(Aii)**; Group “B” (monoesters formed from a ketone body and mid-chain alcohols **(Bi, Bii)** or diesters formed from (*R*)-βHB and mid-chain alcohols and mid-chain carboxylic acids **(Biii)**.

**FIGURE 4:**
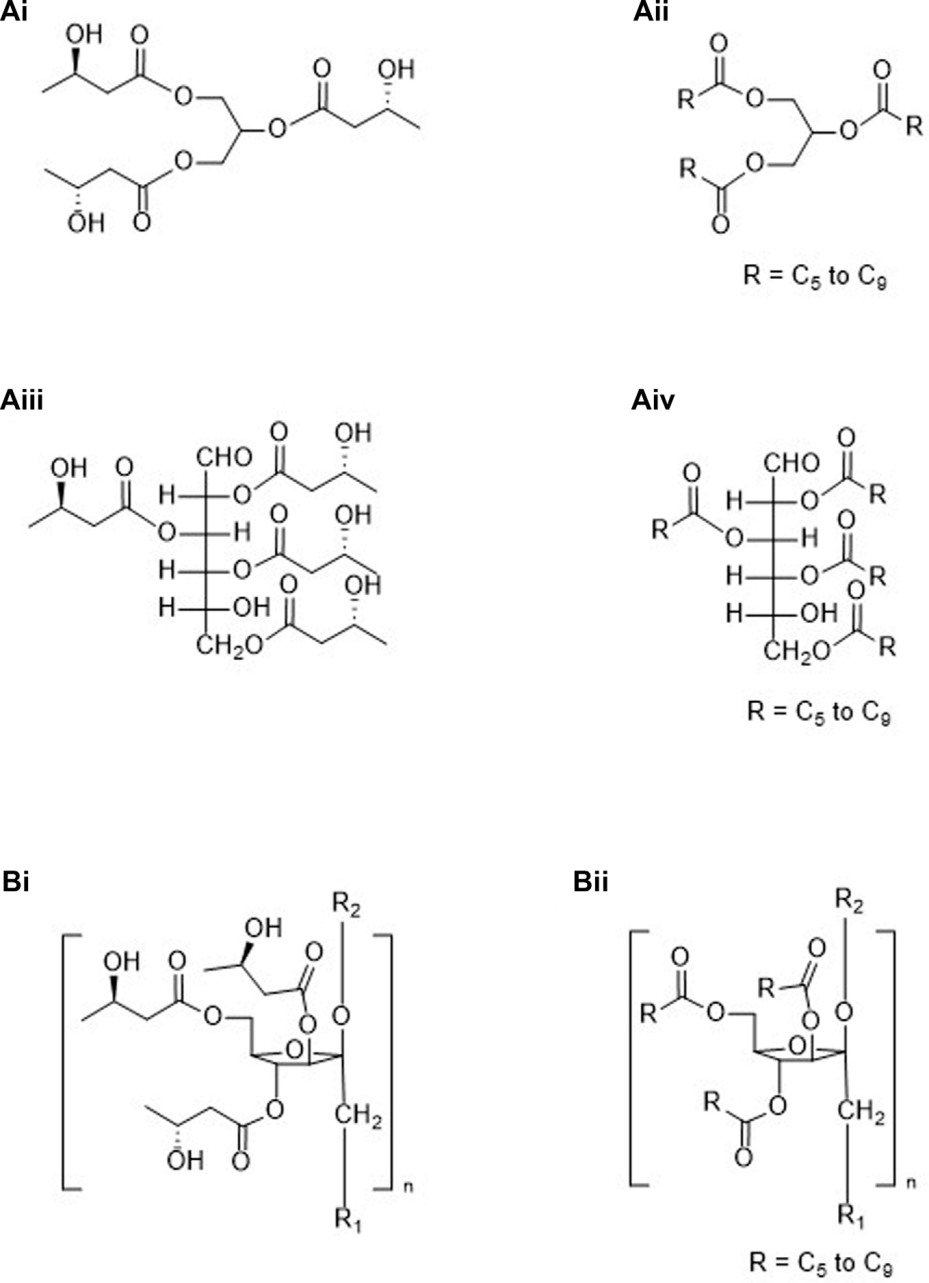
Esters created using ketone bodies plus non-ketogenic compounds: Group “A” (esters formed from a glycerol core with a ketone body **(Ai)** or a pro-ketone body **(Aii)** or a metabolizable polyol core with a ketone body **(Aiii)** or pro-ketone body **(Aiv)**; Group “B” (esters formed from a non-metabolizable polyol and ketone bodies **(Bi)** or pro-ketone bodies **(Bii)**.

Categories 1 through 6 shown below, along with Figures 1–4, are “ketone esters”, or compounds used to synthesize ketone esters, which are independently capable of elevating circulating ketone bodies-those compounds being 1,3-butanediol and mid-chain carboxylic acids and alcohols. Six ketone esters cover the bulk of studies performed to date, those coming from the Brunengraber group, category 3A.ii (Desrouchers et al., 1995), the Veech/Clarke groups, category 3A.i (Clarke et al., 2012), the Hashim group, category 4A.i (Hashim, 2014) and the Buck Institute, categories 2C.iii.2 and 3B.iii (Newman et al., 2017), (Stubbs et al., 2021) along with medium chain triglycerides, category 4A.ii. Immediately obvious from the structures shown in Figures 1–4 are the additional number of compounds possible. Any structure containing a free alcohol or carboxylic acid functional group is capable of further esterification with a ketone body or pro-ketone body. Additionally, polyols may be partially or completely esterified. Structural complexity may lend itself to more favorable pharmacologic or gastrointestinal effects.

Considering the possibilities, and, given the current state of confusion with apparently contradictory experimental outcomes using ketone ester in similar experimental designs, along with the casual use of the term “ketone ester” within the field, it seems prudent to develop and adopt some form of standardized nomenclature. An example categorization is presented below.

Applying unique identifiers to ketone esters, both those currently in use as well as those yet to be developed, could help avoid confusion in reports of studies using these compounds.

### Ketone ester categories (including frequently used pro-ketones)

1. Molecules containing only the ketone bodies βHB and/or AcAc (see Figure 1).
  A. Oligomers of βHB
    i. Linear (Figure 1A).
    ii. Cyclic (Figure 1B).
  Β. βHB oligomers containing a terminal AcAc moiety (Figure 1C).
2. Molecules containing only pro-ketone bodies (See Figure 2).
  A. Compounds converted to ketone bodies by NAD-linked oxidation, e.g., 1,3-BD*
    i. Stereospecific (Figure 2Ai).
    ii. Racemic (Figure 2Aii).
  B. Compounds converted to ketone bodies by β-oxidation, e.g., C_6_–C_10_ carboxylic acids* (Figure 2B).
  C. Esters created from pro-ketone bodies
    i. C_6_–C_10_ carboxylic acids (β-oxidation) and C_6_–C_10_ alcohols (NAD-linked and β-oxidation) monoesters.
      1. Homo esters (Figure 2Ci, *n* = n’)
      2. Mixed esters (Figure 2Ci, *n* ≠ n’)
    ii. C_6_–C_10_ carboxylic acids and diol, e.g., 1,3-BD (NAD-linked oxidation) monoesters.
      1. Stereospecific (Figure 2Cii.1).
      2. Racemic (Figure 2Cii.2).
    iii. C_6_–C_10_ carboxylic acids and diol, e.g., 1,3-BD (NAD-linked oxidation) diesters.
    1. Stereospecific
      a. Homo esters (Figure 2Ciii.1, *n* = n’)
      b. Mixed esters (Figure 2Ciii.1, *n* ≠ n’)
    2. Racemic
      a. Homo esters (Figure 2Ciii.2, *n* = n’)
      b. Mixed esters (Figure 2Ciii.2, *n* ≠ n’)
3. Molecules containing both pro-ketone bodies and ketone bodies (See Figure 3).
  A. Ketone bodies plus compounds converted to ketone bodies by NAD-linked oxidation
    i. Stereospecific.
      1. Monoester (Figure 3Ai).
      2. Diester.
      3. Mixed diester.
    ii. Racemic.
      1. Monoester.
      2. Diester (Figure 3Aii).
      3. Mixed diester.
  B. Ketone bodies plus compounds converted to ketone bodies by β-oxidation or by both NAD-linked and β-oxidation
    i. Monoesters of AcAc (Figure 3Bi).
    ii. Monoesters of (*R*)-βHB (Figure 3Bii).
    iii. Diesters of (*R*)-βHB.
      1. Homo-acid and alcohol having equal carbons (Figure 3Biii, *n* = n’)
      2. Mixed-acid and alcohol having unequal carbons (Figure 3Biii, *n* ≠ n’)
4. Esters created using ketone bodies or pro-ketone bodies plus non-ketogenic compounds-partial or complete esterification possible (See Figure 4).
  A. Metabolizable
    i. Glycerides (Figure 4Ai).
    ii. MCTs, medium chain triglycerides (Figure 4Aii).
    iii. Mono-, di- and polysaccharides (Figure 4Aiii, glucose Fisher projection).
    iv. Mono-, di- and polysaccharides esterified to mid-chain carboxylic acids (Figure 4Aiv).
  B. Non-metabolizable
    i. Inulin and a ketone body (Figure 4Bi, partial structure).
    ii. Inulin and a pro-ketone body (Figure 4Bii, partial structure).
5. Esters created using ketogenic amino acids and (*R*)-βHB.
6. Esters created using ketogenic amino acids and pro-ketone bodies.
  A. Esterifying section 2 alcohols to ketogenic amino acids.

*While not esters, 1,3-butanediol and mid-chain carboxylic acids are pro-ketone bodies and all are integral in many of the “ketone esters” created to date and hence are included here.
